# Adaptive Performance in Healthcare: The Role of Collaboration‐Oriented Leadership, Work Engagement, Group Identification, and Change Predisposition

**DOI:** 10.1155/jonm/4210701

**Published:** 2026-09-01

**Authors:** Chiara Panari, Luciano Ferrari, Alice Bonini

**Affiliations:** ^1^ Department of Economics and Management, University of Parma, Parma, Italy, unipr.it; ^2^ Department of Medicine and Surgery, University of Parma, Parma, Italy, unipr.it

**Keywords:** adaptive performance, change predisposition, collaboration, collaboration-oriented leadership, group identification, health care, human resources, nursing management, work engagement

## Abstract

**Aim:**

This study aims to investigate the relationship between a leadership approach oriented toward collaborator involvement and adaptive performance, with work engagement and group identification as motivational mediators. Personal change predisposition was examined as a moderator between collaboration‐oriented leadership, work engagement, and adaptive performance.

**Background:**

Adaptive performance is essential for maintaining high‐quality patient care in rapidly changing healthcare settings. Although collaborative leadership has been associated with several positive organizational outcomes, the mechanisms through which it supports healthcare professionals’ adaptive performance remain unclear.

**Methods:**

The authors conducted a cross‐sectional study in a hospital in northern Italy. An online questionnaire was administered to 418 healthcare professionals, and more than half of the respondents were nurses.

**Results:**

Collaboration‐oriented leadership may support adaptive performance positively, both directly and indirectly, through work engagement and group identification. Change predisposition had a moderating effect only between leadership and adaptive performance and between leadership and work engagement, suggesting that collaboration‐oriented leadership is more effective in enhancing adaptivity and engagement when healthcare professionals exhibit lower levels of change predisposition.

**Conclusion:**

The findings highlight the importance of adopting a bottom–up leadership approach by actively involving and supporting team members to promote adaptive strategies in organizational contexts and situations characterized by greater resistance to change of human resources.

**Implications for Nursing Management:**

In terms of practical implications, nurse managers and care‐unit leaders could increase staff work engagement and the sense of group identification by providing additional supports to the professionals who are less predisposed to embrace organizational changes and are more resistant to innovation.

## 1. Introduction

Hospital healthcare professionals are constantly involved in rapid and significant changes that affect their organizational roles and work activities [1, 2]. These changes can be due to the external environment or may depend on internal organizational needs. For example, healthcare organizations must deal effectively with unpredictable work situations and adapt to challenges and constraints caused by emergencies and crises. The use of new technology and the need to manage highly diversified and specific patient care and treatment, which require individualized approaches based on the user and the pathology, are other examples of a great capacity to adapt by individual healthcare professionals [3]. Moreover, political reforms or initiatives and changes in healthcare legislation often require an internal redefinition of procedural processes involving all hospital staff, both individually and at the team level [4].

All these challenges have significantly altered the nature of healthcare work, placing growing demands on physicians, nurses, and allied health professionals. Within this dynamic context, the ability of healthcare professionals to adapt effectively to changing work conditions has become a critical determinant of care quality, patient safety, and organizational resilience. Consequently, adaptive performance (AP), defined as the capacity to adjust behaviors, skills, and strategies in response to novel, uncertain, or rapidly changing demands [5–8], has emerged as a key outcome in contemporary healthcare organizations. For these reasons, it becomes necessary to investigate adaptive behaviors in response to changing working conditions and identify effective organizational strategies to address uncertainty, especially for professionals working in health sectors.

## 2. Literature and Hypotheses

### 2.1. AP and Organizational Changes

Allworth and Hesketh first introduced AP as a separate construct from task and contextual performance [9], and the rapidity and unpredictability of changes in the labor market have increased interest in the ability of workers to respond to changes and implement AP. Therefore, the construct of AP has grown as an alternative to traditional concepts of performance, highlighting the ability of employees to change their behavior according to the needs of each new environment and to proactively respond to changes in job situations [10, 11]. The behaviors performed could include addressing emergencies, solving problems creatively, training and learning efforts, interpersonal adaptability, and managing work stress [5, 12].

Griffin et al. [6] identified three dimensions: proficiency, adaptivity, and proactivity, which act on three different levels: individual, group and organizational, as key elements for responding to changes.

In healthcare, there is a growing awareness of the need for healthcare professionals to face organizational demands with adaptive and proactive strategies [13]. The AP of healthcare professionals refers to the ability of individuals and teams to go beyond simply following established protocols (task performance) to act with the flexibility and responsiveness needed to manage unpredictable situations, learn skills, and continuously improve patient care [14]. Healthcare professionals operate in a complex and dynamic network of individuals in which the environment could rapidly change; the COVID‐19 pandemic, for example, clearly demonstrated the need to be extremely adaptable, take new roles, reorganize teams, operate with limited resources, and be innovative and creative to cope with emergencies [15]. Moreover, encouraging adaptive behaviors can lead to greater job satisfaction and a sense of mastery, as operators feel more confident in their ability to handle job demands during stressful conditions [16].

Moreover, hospital work is now based on the coordination of interdisciplinary teams, in which health professionals from various disciplines must align their actions to manage the increasing complexity of tasks and provide efficient and effective healthcare [17]. As a result, scientific research is increasingly focusing on how to promote, support, and maintain team adaptability through group interaction [18].

In line with the needs explained, this study aimed to examine AP at the group level in a healthcare organization and its antecedents, focusing particularly on collaboration‐oriented leadership (CL) and considering two possible mediating variables related to the motivational process: work engagement (WE) and group identification (GI). The authors also consider personal predisposition to change as an individual moderating variable.

### 2.2. CL and AP

Recent literature has increasingly emphasized the role of leadership, as an organizational factor, in fostering adaptive behaviors within healthcare organizations [19], particularly in contexts characterized by uncertainty, complexity, and continuous change [19, 20].

A recent meta‐analysis and systematic review demonstrated the positive influence of follower‐centered leadership in enhancing AP [21]. Leadership approaches based on team members’ relationships seem to be more effective in achieving new goals of patient care [22, 23] and addressing the increasing complexity of tasks and rapid innovations that mean being involved in everyday change processes that require the use of different professional skills. CL refers to collaborate leadership style identified by the model of competitive values [24]; it is a leadership approach based on a model of organizational culture defined as Clan [25] that focuses on people cohesion and team motivation to work with each other. Specifically, it concerns a bottom–up managerial strategy that improves the satisfaction and involvement of collaborators by fostering their involvement and participation, by promoting open communication and coordination, and by creating organizational conditions that support adaptability and flexibility [26]. AP can be understood not only as individual factors but rather as the outcome of relational and contextual dynamics embedded within the work environment [6, 10, 11]. Healthcare professionals are frequently required to respond rapidly to evolving clinical, organizational, and technological demands. Evidence indicates that inadequate staff involvement in the planning and implementation of management‐led organizational change may foster resistance and change fatigue. In contrast, healthcare professionals seem to support successful change initiatives if they have the opportunity to influence them, if they are prepared for the changes and their consequences, and finally, if they value the changes [4].

When participative leaders involve employees in decision‐making and problem‐solving, employees feel encouraged, supported, and responsible for making innovative new contributions [27]. In this perspective, leaders contribute not only by directing behaviors but also by shaping supportive social climates that encourage engagement, trust, and coordinated responses to organizational challenges. Such relational mechanisms appear especially relevant in healthcare organizations, where adaptive functioning often depends on effective teamwork and collective sensemaking processes [28].

Starting from these premises, our first hypothesis (H1) is that CL approach is directly and positively related to AP.

### 2.3. The Mediational Role of WE

The literature has highlighted the presence of many factors that can mediate the relationship between leadership and AP, including WE and dynamics related to the workgroup [21].

WE, defined as a “*positive, fulfilling, work-related state of mind characterized by vigor, dedication and absorption*” [29], is one of the antecedents of AP [8] and is one of the most cited motivational resources that increase employee well‐being [30, 31]. Highly engaged employees are more likely to invest additional effort in their jobs and are better prepared to manage the dynamics of change [32]. Breevaart et al. [33] reported that workers who feel engaged in their work focus more on their daily performance and dedicate their energy and attention to their tasks, demonstrating greater awareness of their work. This awareness enables them to be better prepared to face new external challenges and changes.

Additionally, the job demands‐resources (JD‐R) model of WE [34, 35] conceptualizes how job characteristics promote employees’ motivation, which in turn influences their job performance. In this sense, several longitudinal studies have shown that WE is driven primarily by work‐related factors such as leadership [36, 37]. In particular, a leadership style that enables team members to coordinate effectively with one another, and encourages them to participate in organizational processes, increases followers’ satisfaction and motivation [38]. Leaders can satisfy their followers’ needs for autonomy, competence and relatedness by facilitating involvement, strengthening their skills, and connecting them, respectively [39]. In this sense, CL, which focuses on developing people in terms of participation and personal growth [26], can satisfy the needs that underlie the motivational processes that trigger engagement.

Starting from these assumptions, our second aim was to examine how collaborate leadership indirectly was associate to AP through the mediational role of WE. Our second hypothesis (H2) is that WE has a mediating role between CL and AP.

### 2.4. The Mediational Role of Social Identification

CL approaches differ from traditional top–down managerial and neo‐charismatic, which view the leader as an influent and charismatic figure that is alone capable of being a change guide for his/her followers (e.g., [40]) and moves toward a less hierarchical model of governance that values the involvement of team members as a form of adaptation to healthcare changes [41]. While leader support and transformational leadership are organizational antecedents of individual AP [8, 32, 42, 43], in the healthcare sector, interest in a collective approach to the study of AP is increasing due to the strong interdependence of roles and greater interest in group dynamics and teamwork [44]. This calls into question the social cure approach [45], which represents the application of the principles of social identity theory [46] and self‐categorization theory [46], starting from the assumption that groups to which people belong are important determinants of people’s health and productivity [45]. GI, which refers to the extent to which people feel tied to their group, becomes a source of motivation to work hard and achieve goals with positive influences on well‐being [47]. High levels of organizational identification are associated with increased employee commitment, motivation, and collaboration, leading to more effective and cohesive teamwork [48]. Shared membership can indeed improve the perception that social support is available, affecting how people appraise their ability to cope with stressful events and challenges [49, 50]. Group‐oriented followers focus on the collective interests of their group, helping other members and suggesting ideas for improvement and adaptation to change [51]. Leadership can act as a catalyst for this GI process. Leaders who work to represent, advance, create, and embed an identity shared by members of a particular group enhance GI [50, 52]. More specifically, leaders who reinforce team members’ collective sense of “us” can affect team members’ confidence by strengthening an internalized and shared group identity [53]. Men and Yue [54] emphasized that managers can enhance employees’ sense of belonging and alignment with corporate objectives via effective communication, promoting transparency and encouraging active participation in group decision‐making.

Building on these premises, we propose that leaders’ and managers’ ability to communicate effectively and foster a shared identity is a key determinant of GI, which, in turn, increases the likelihood that their efforts to promote followers’ adaptability will succeed. Specifically, we hypothesized (H3) that GI has a mediating role between CL and AP.

We hypothesized two complementary pathways through which collaborate leadership may be related to adaptation in healthcare professionals. The first pathway is grounded in the JD‐R model and reflects an individual motivational process in which CL represents an important job resource that may enhance employees’ psychological energy, dedication, and involvement in work activities, thereby increasing WE and facilitating AP in dynamic organizational contexts [55, 56].

The second pathway is based on social identity theory and reflects a collective relational process; specifically, CL may strengthen employees’ sense of belonging, shared identity, and alignment with collective goals, thereby fostering GI [46, 49]. In turn, stronger identification with the team may facilitate AP characterized by high levels of interdependence and intensive teamwork demands.

### 2.5. The Moderating Role of Change Predisposition

As early as 2000, LePine et al. [57] characterized people open to new experiences as individuals with strong intellectual curiosity who typically seek less conventional experiences. The authors underlined how being open to experience makes a person more inclined to participate in new types of self‐monitoring that are necessary for learning new skills in changing working environments and using creativity, which is very important for adaptability. In a study by Ogunfowora et al. [58], researchers reported a positive relationship between openness to experience and AP. Openness to change and openness to new experiences are predictors of work role adaptive and proactive behaviors [6, 59].

Some studies have shown that openness to experience is a significant predictor of engagement [60, 61] and that willingness to change is linked to job crafting, particularly by stimulating strategies of seeking resources and challenges [62].

Effective leadership is closely related to the success of transformation processes within companies. In healthcare contexts, the literature has shown that bottom–up leadership approaches are especially effective for addressing personal change resistance, which involves negative actions aimed at sabotaging organizational change initiatives [63].

Leadership support in building a shared vision, translating policy into objectives, providing coaching support for staff development, communicating persuasively, and encouraging the active participation of members provides structural readiness for change [64].

In healthcare contexts, the literature has shown that bottom–up leadership approaches are particularly effective in addressing resistance to personal change, which involves negative actions aimed at sabotaging organizational change initiatives [63]. A study on nurses carried out by Naami et al. [65] revealed that openness to new experiences is strongly linked to adaptivity, much more than other personal characteristics are.

Building on these premises, we expected that change predisposition, a dimension of change acceptance reflecting the perception of change as an opportunity for growth [66], would act as an individual protective factor against change‐related challenges and as an antecedent of AP. Specifically, professionals with a high predisposition to change may already possess internal motivational and cognitive resources that facilitate adaptation independently of leadership influence; in contrast, people with a lower predisposition to change may rely more strongly on external relational and organizational resources provided by leadership behaviors, such as communication, support, participation, and psychological safety, in order to cope effectively with change and uncertainty and overcome resistance when changes are perceived as threatening.

Our fourth hypothesis is that change predisposition, as a personal resource, moderates the relationship between CL and AP (H4a), between CL and WE (H4b), and between WE and AP (H4c).

**FIGURE 1:**
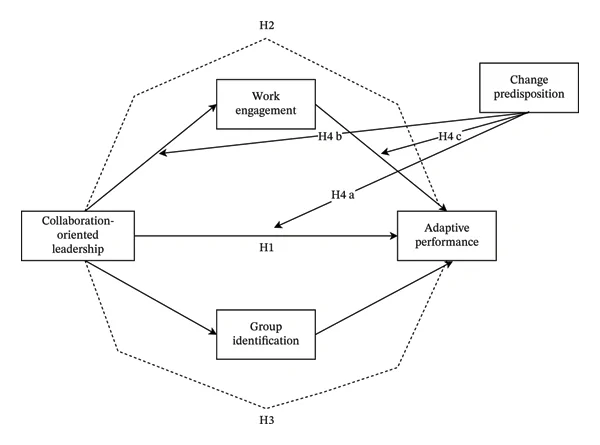
Conceptual model of the study.

The study aims to explore how healthcare organizations can foster AP among professionals working in increasingly complex and rapidly changing contexts, focusing on how CL can indirectly promote such performance through organizational and individual mechanisms, such as WE, care unit identification, and change predisposition. The complete conceptual model of the study is shown in Figure 1.

## 3. Materials and Methods

### 3.1. Instruments and Procedures

To test our hypotheses, we conducted a quantitative cross‐sectional study in a large hospital locate in Northern Italy. The Hospital Joint Committee of Equal Opportunity and hospital management were involved in promoting the study among healthcare staff. An anonymous voluntary questionnaire was administered from March to November 2022 via the Qualtrics online survey platform. The survey questions were addressed to hospital health personnel and were adopted from previously validated national and international measurement scales.

### 3.2. Sample Description

The hospital healthcare professionals were invited to participate voluntarily in an online survey distributed internally within the organization. A total of 901 individuals initially accessed and completed the questionnaire platform. Among these, 198 respondents were excluded because they did not answer any questionnaire items. Of the remaining participants, 418 healthcare professionals provided sufficiently complete responses and were, therefore, included in the final analyses, corresponding to approximately 60% of the eligible respondents who actively engaged with the survey. The average age of the participants was 44.40 years (SD 10.18; range from 23 to 67 years). A total of 78.7% were female (n. 329), and 21.3% were male (n. 89). With respect to organizational role, the majority were nurses (54.5% of the total respondents), followed by physicians (26.3%), social health workers (11.13%), and other technical health professionals (7.3%).

### 3.3. Measurements

To detect CL aspects, we chose the manager behavioral instrument (MBI) [26], which examines leadership behavior from the organizational culture point of view via the theory of the competing values framework [67]. The participants responded to three items expressing their degree of agreement or disagreement regarding the behavior adopted by their immediate supervisor (physician or nurse). This scale detects the “Clan” cultural dimension of managerial orientation.

WE was measured via Italian validation by Balducci and Colleagues [68] of the Utrecht WE Scale (UWES) [31], which includes nine items of three dimensions of WE: vigor, dedication, and absorption.

Change predisposition was measured with the Italian four‐item Di Fabio Acceptance of Change Scale [66]. The items were contextualized on changes that occur in the workplace.

We adopted the framework proposed by Griffin, Neal, and Parker [6], which distinguishes work role performance according to different levels: individual, team, and organization to detect participants’ AP at group level. Since, in the present study, AP was conceptualized and measured at the individual level, we chose the three items from Griffin, Neal, and Parker’s scale [6], which focuses on interpersonal adaptivity behaviors of work role performance, specifically on the degree to which the person feels able to cope with changes at work at the group level.

Finally, GI was measured by referring to hospital care unit identification via the single item “being part of my operational unit is an important part of the image I have of myself” [69].

Analyses were conducted via SPSS software (Statistics for Data Analysis, Version 29) for descriptive, reliability, and correlational analyses, while the moderated mediation analyses were conducted via Jamovi statistical analysis software (Version 2.4.14).

All variables included in the study were measured on a seven‐point Likert scale and analyzed at the individual level.

## 4. Results

### 4.1. Hypotheses Test and Assumption Verification

A multiple moderated mediation statistical model was tested to explore the effect of the independent variable (CL) on the dependent variable (AP) through two mediating variables in parallel (M1: WE and M2: group identification—GI), conditioned by a moderating variable (change predisposition). In accordance with the theoretical model, the multiple mediation process is modulated by the moderating variable in the relationships (a) between CL and AP, (b) between CL and WE, and (c) between WE and AP.

First, a linear correlation analysis was performed, revealing significant positive correlations among the variables considered in the model (see Table 1).

**TABLE 1:** Correlations among variables.

	Mean (±SD)	*α*	1	2	3	4	5
1. Collaboration‐oriented leadership	3.97 (1.8)	0.96	1				
2. Work engagement	4.57 (1.34)	0.79	0.29^∗∗^	1			
3. Group identification	5.73 (1.50)	—	0.27^∗∗^	0.49^∗∗^	1		
4. Adaptive performance	5.32 (1.27)	0.97	0.32^∗∗^	0.44^∗∗^	0.32^∗∗^	1	
5. Change predisposition	4.79 (1.33)	0.92	0.24^∗∗^	0.41^∗∗^	0.23^∗∗^	0.60^∗∗^	1

*Note: n* = 418; all variables are significant ^∗∗^ *p* < 0.01.

### 4.2. Verification of the Mediation Effects

In the context of the multiple mediation analysis conducted, the results reveal the specific importance of the mediating variables through which leadership may be related to group AP.

The analysis of the individual components of the mediation paths reveals direct and significant relationships (see Table 2). CL positively predicted both WE (*β* = 0.217, *p* < 0.001) and GI (*β* = 0.269, *p* < 0.001), which in turn were positively associated with AP (*β* = 0.147, *p* = 0.001 and *β* = 0.099, *p* = 0.021, respectively). Significant indirect effects emerged through WE and organizational identification.

**TABLE 2:** Standardized direct, indirect and total effect of collaboration‐oriented leadership (CL), work engagement (WE), group identification (GI) and adaptive performance (AP).

Type	Effect	*B*	SE	95% C.I. (a)		*β*	*z*	*p*	
				Lower	Upper			
Indirect	CL ⇒ WE ⇒ AP	0.0344	0.0149	0.00494	0.0634	0.0349	2.31	< 0.021	
	CL ⇒ GI ⇒ AP	0.0268	0.0135	−7.84*e* − 4	0.0537	0.0271	1.98	0.048	
	
Component	CL ⇒ WE	0.2337	0.0471	0.14115	0.3259	0.2383	4.96	< 0.001	
	WE ⇒ AP	0.1473	0.0552	0.03817	0.2544	0.1466	2.67	0.008	
	CL ⇒ GI	0.2692	0.0493	0.17296	0.3661	0.2692	5.46	< 0.001	
	GI ⇒ AP	0.0994	0.0451	0.01272	0.1895	0.1008	2.20	0.028	
	
Direct	CL ⇒ AP	0.1430	0.0387	0.06778	0.2196	0.1450	3.69	< 0.001	
	
Total	CL ⇒ AP	0.1915	0.0413	0.11031	0.2723	0.1915	4.63	< 0.001	

*Note: N* = 418. Standardized coefficients (*β*) are reported. Indirect effects were estimated using bias‐corrected bootstrapping with 5000 resamples.

The analysis showed the significant role of mediating variables (WE and GI) in transferring the impact of CL on AP.

The mediation path CL ⟶ WE ⟶ AP has a significant effect (*β* = 0.0349, *p* 0.021), underscoring the importance of WE as a conduit through which CLwas associated with group AP. Similarly, the mediation path CL ⟶ group identification ⟶ AP presents a slightly less prominent but still significant impact (*β* = 0.0271, *p* = 0.048), indicating that organizational identification also contributes indirectly to group AP.

Direct and total effects underscore the overall relationship between CL and AP; the direct effect of collaborate leadership on AP is significant (*β* = 0.1450, *p* < 0.001), highlighting its positive and direct role in enhancing group performance.

**FIGURE 2:**
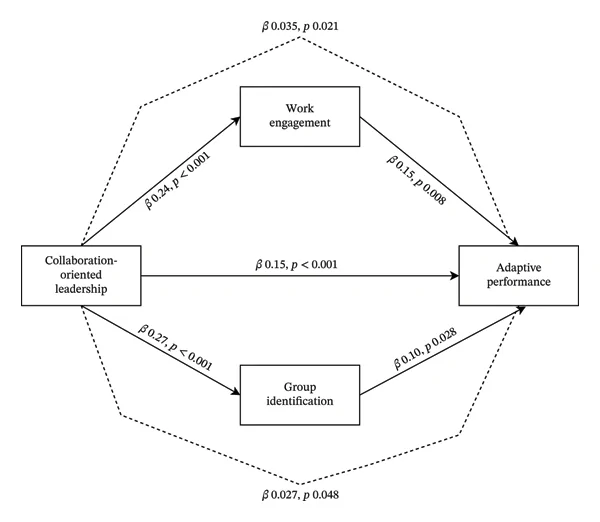
The moderation model tested.

Furthermore, the total effect of CL on AP is also significant (*β* = 0.1915, *p* < 0.001). The expected model is presented in Figure 2.

### 4.3. Verification of the Moderation Effects

The moderated mediation model showed good explanatory power, explaining 21.4% of the variance in WE (*R* ^2^ = 0.214, *F* = 37.50, *p* < 0.001) and 44.3% of the variance in AP (*R* ^2^ = 0.443, *F* = 54.50, *p* < 0.001).

### 4.4. Moderation Effects of Change Predisposition on the Relationship Between CL and WE

Results indicated that CL (*β* = 0.217, *p* < 0.001) and change predisposition (*β* = 0.335, *p* < 0.001) were both positively associated with WE and the interaction between CL and change predisposition was statistically significant (*β* = −0.093, SE = 0.043, *z* = −2.16, *p* = 0.031), indicating that change predisposition reduces the effect of CL on WE.

**FIGURE 3:**
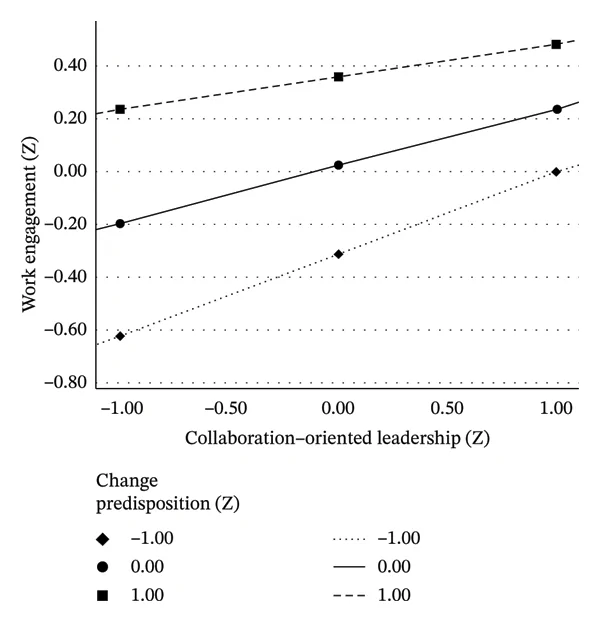
Interactions between change predisposition and collaborate leadership and work engagement.

Conditional effects analysis revealed that the positive influence of CL on WE progressively decreased as change predisposition increased. Specifically, the effect was strongest when change predisposition was low (mean −1 SD, *β* = 0.333, *p* < 0.001), remained significant at means levels (*β* = 0.238, *p* < 0.001), and was weaker, although still significant, when change predisposition was high (mean + 1 SD, *β* = 0.144, *p* = 0.026). Figure 3 represents the interaction effect between change predisposition, CL, and WE.

This suggests that CL is much more effective in influencing WE when professionals exhibit lower levels of change predisposition; conversely, professionals with high levels of change predisposition, who already exhibit a strong personal predisposition to innovation and organizational change, tend to be less influenced by CL.

### 4.5. Moderation Effects of Change Predisposition on the Relationship Between CL and AP

A significant interaction was also found on the relationship between CL and change predisposition in predicting AP (*β* = −0.083, SE = 0.034, *z* = −2.44, *p* = 0.015). This result indicates that change predisposition moderates the direct relationship between CL and AP.

**FIGURE 4:**
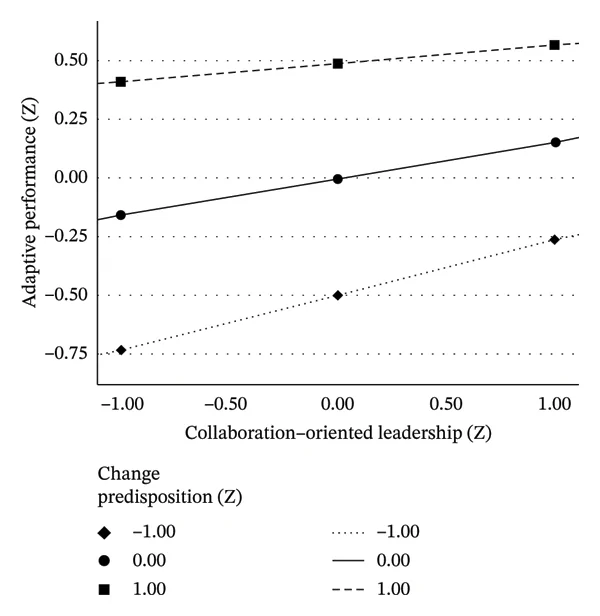
Interactions between change predispositions on collaborate leadership and adaptive performance.

The conditional direct effects showed that CL and AP have a stronger relationship when change predisposition was low (mean −1 SD *β* = 0.230, *p* < 0.001). As shown in Figure 4, the effect remained significant at average levels of change predisposition (*β* = 0.145, *p* < 0.001) but became nonsignificant when change predisposition was high (mean + 1 SD, *β* = 0.061, *p* = 0.173).

This result suggests that a low level of change predisposition may amplify the positive relationship between CL and group AP, underscoring the importance of considering individual differences in leadership effectiveness within dynamic team settings. Moreover, in the presence of high levels of change predisposition, the relationship between CL and AP becomes less significant than professionals with medium or lower change predisposition propensity; it underscores the need to balance individual traits of change predisposition and leadership function in such a way as to promote teamwork in groups of highly adaptable and high‐performing professionals.

### 4.6. Moderation Effects of Change Predisposition on the Relationship Between WE and AP

An additional hypothesis examined the effectiveness of readiness for change as a factor capable of influencing the relationship between WE and AP. The observed interaction does not exhibit significant values, indicating that the relationship between WE and AP is independent of change predisposition (*β* = 0.058, SE = 0.032, *p* = 0.077).

The table below (Table 3) summarizes the results of the hypotheses tested.

**TABLE 3:** Hypothesis‐testing summary.

Hypothesis	*β*	95% bootstrap CI		*p*	Supported
		Lower	Upper		
H1: Collaboration‐oriented leadership ⟶ group adaptive performance	0.1915	0.1140	0.2689	< 0.001	Yes
H2: Collaboration‐oriented leadership ⟶ work engagement ⟶ group adaptive performance	0.0349	0.0049	0.0634	0.021	Yes
H3: Collaboration‐oriented leadership ⟶ care unit identification ⟶ group adaptive performance	0.0271	0.0008	0.0537	0.048	Yes
H4a: Change Predisposition moderates the relationship between leadership and Adaptive performance	−0.0828	−0.1568	−0.0088	0.028	Yes
H4b: Change Predisposition moderates the relationship between leadership and Work engagement	−0.0929	−0.1770	−0.0084	0.031	Yes
H4c: Change Predisposition moderates the relationship between Work engagement and Adaptive performance	0.0576	−0.0062	0.1215	0.077	No

## 5. Discussion

This study aimed to provide insight into the relationship between CL and group AP. In particular, the results highlighted that CL affects AP both directly and indirectly, confirming hypotheses H1, H2, and H3. Extensive research has linked various leadership approaches to AP, particularly those characterized by greater flexibility and lower control, which acknowledge employees’ needs and encourage their active involvement [8, 20, 70]. A leader who stimulates employees to learn and facilitates knowledge exchange and collaboration, encouraging bottom–up initiatives, can help employees recraft their roles to accommodate externally driven changes [71].

Moreover, many studies have highlighted some personal and organizational factors that mediate the relationship between leadership and AP [32, 72, 73]. Specifically, in this study, we considered both the personal motivational process of WE and, at the organizational level, the GI. The results confirm hypothesis H2, showing the mediational role of WE. As the literature has shown [74], a leadership approach oriented toward collaboration and the development of positive interactions with employees, along with encouraging participation, provides them with greater intrinsic motivation, positive energy, and commitment to change. By involving employees in decision‐making and problem‐solving, CL empowers them to contribute creatively, fostering a sense of encouragement and support. The motivational processes activated by WE are, therefore, the basis for AP especially that which is linked to a proactive attitude of the work group toward the required changes. Engaged employees are more proactive, take the initiative to collaborate with others, and are committed to achieving high‐quality performance. Furthermore, in line with other studies [75], WE fosters attitudes toward work that motivate employees to assume new responsibilities as part of their job, which can translate into proactive behavior in the face of change.

The study also revealed that GI played a pivotal role in mediating the relationship between CL and group AP, supporting hypothesis H3. Other researchers [76] have emphasized that the way followers feel and define themselves within a group (i. e., GI) mediates the main effect of leadership on performance. According to the social cure approach [45], GI represents collective identity orientation, illustrating how group members define themselves in terms of the values, goals, attitudes, and behaviors they share with other group members. Employees who closely align with their organization’s identity are more prone to exhibit behaviors that support the organization and contribute positively to organizational productivity [77]. Supervisory support and opportunities to participate in decision‐making on important issues can strengthen employees’ sense of belonging. GI becomes particularly salient when collaborative leaders help followers make sense of organizational change and foster a shared collective identity.

Conversely, when identification with the team is low, individuals feel a sense of detachment from the organization and thus may not feel part of organizational change. Collaborative and supportive leaders create a favorable environment by addressing team members’ needs and valuing their perspectives, thereby establishing the conditions needed to foster adaptive behavior within the group. Shared decision‐making empowers employees, leading to increased job satisfaction and a stronger sense of belonging [78], and leader bottom–up communication is essential for the members’ formation of a shared social identity [79]. Furthermore, when employees are involved in decision‐making, they are less likely to feel threatened by organizational change, and they are more likely to deal with changes in their work group and learn new skills or take on new roles to cope with new ways their work group is required to incorporate.

In this sense, empirical support has been found for the role of GI in predicting high levels of creativity [80, 81].

Finally, a particularly noteworthy finding concerns the moderating role of change predisposition; partially confirming hypothesis H4, the individual predisposition to change had a moderating effect on both the process that involved CL and AP (H4a) and CP and WE (H4b). Contrary to what was hypothesized in H4c, this personal variable does not appear to contribute to the impact of WE on AP. Results suggest that CL exerts a stronger influence on WE and AP among healthcare professionals with lower change predisposition and suggest that leadership helps to reduces resistance to change by promoting the motivational process of WE and the adoption of adaptive behaviors.

Findings can be interpreted through the JD‐R framework, which posits that organizational and personal resources may operate in complementary or compensatory ways [35, 82]. Employees who are naturally open to change already possess an important personal resource that facilitates adaptation to uncertain and evolving work environments, and consequently, they may rely less on leadership support. In contrast, individuals with a lower predisposition for change may benefit more strongly from leadership behaviors that foster communication, participation, support, and psychological safety, thereby compensating for lower personal resources and facilitating adaptation [19, 55]. The influence of CL on followers’ motivation increases when personal openness toward change is low, and this style would seem particularly important when collaborators see change as an experience that brings insecurity and disorientation and not as an opportunity for growth.

Another relevant finding concerns the absence of a significant moderating effect of change predisposition on the relationship between WE and AP. This result suggests that WE may function as a relatively robust motivational state that supports adaptive behavior across different levels of openness to change. Consistent with previous research, engaged employees tend to invest physical, cognitive, and emotional resources in their work, which promotes proactive and adaptive responses regardless of individual differences in change readiness [83, 84]. Therefore, change predisposition appears to have a less evident role in determining how engagement translates into adaptive behaviors.

Supportive leadership is important for bringing out the best in teams, particularly during times of change [85], and this suggests that leadership focused on human resources may contribute to increase followers’ adaptivity through the development of their intrinsic motivation [86].

## 6. Conclusions

These findings emphasize the importance of CL in promoting group AP, revealing both the mediated mechanisms through WE and organizational identification, as well as the direct impact of leadership on group performance. Even in a very hierarchical context, such as healthcare organizations, leadership based on horizontal integration processes favors an adaptive approach by the team for dealing with emerging change, preventing forms of resistance. The CL can, therefore, encourage openness toward new experiences because he or she promotes clear communication, makes collaborators aware of why things are done, and gives them increased control over their future and changes in tasks. Moreover, by showing good listening skills and giving team members the opportunity to take part in team activities, discussions, and decisions, leaders can promote team identification. This feeling of belonging makes the process of adapting to change safer and less threatening.

### 6.1. Implications for Nursing Management

This study has many implications for the development of a managerial culture strongly based on horizontal processes, even in very hierarchical contexts such as the healthcare organizations involved in the survey. Leadership that is too directive and based on a top–down approach is very ineffective in contexts of change. Therefore, healthcare organizations should recognize that successful change and AP largely depend on how change is perceived.

Findings show that the strength of relationships may depend on individual differences in attitudes toward change. In healthcare settings characterized by the continuous implementation of new technologies, clinical guidelines, and organizational models, openness to change is a salient individual factor. Healthcare professionals who are more open to change tend to perceive change as an opportunity for learning and improvement rather than as a threat, but the positive effect of a leadership approach oriented towards cooperation, support, and enhancement of collaborators’ professional skills is higher when levels of change predisposition are low, showing that a leadership style oriented toward clear communication and a clear understanding of fears and resistance to change can help create the vision and motivation to adopt new ways of doing things.

This suggests that leadership development programs in healthcare should, therefore, move beyond traditional hierarchical models to incorporate training on collaborative competencies, such as inclusive leadership practices, conflict management, and facilitation of interprofessional teamwork.

Indeed, the role of care unit identification underscores the importance of reinforcing a shared professional identity within multidisciplinary healthcare teams. Practical actions may include the promotion of team‐based goals, interprofessional training, and structured opportunities for reflection and learning at the team level. Strengthening GI can facilitate coordination, knowledge sharing, and collective problem‐solving, all of which are critical for AP in complex care pathways.

From a practical perspective, the findings suggest several actions that nurse managers and healthcare leaders can implement to promote AP in increasingly complex and changing healthcare environments. First, leadership development programs should emphasize collaborative communication skills, active listening, and participatory decision‐making practices that encourage healthcare professionals to contribute to problem‐solving and change‐related initiatives. Creating opportunities for regular team reflection and debriefing sessions may also facilitate collective learning, knowledge sharing, and adaptation to evolving clinical and organizational demands. Second, managers should promote interprofessional collaboration and learning by encouraging exchanges among nurses, physicians, and other healthcare professionals. Such practices may strengthen both WE and identification with the care unit, fostering a shared sense of purpose and collective responsibility for patient care outcomes. Finally, the present findings suggest that CL is especially beneficial for employees with lower predisposition to change; staff members who may feel uncertain or resistant during organizational transitions could be supported through coaching, mentoring programs, and others opportunities to gradually develop confidence in managing change. By combining relational support with participatory leadership practices, healthcare organizations may enhance both employee well‐being and AP, ultimately contributing to higher quality and more resilient healthcare services.

### 6.2. Limitations and Future Directions

This research has several limitations. The first is regarding the cross‐sectional nature of the study, and the use of a convenience sample drawn from a single healthcare organization may limit the external validity and transferability of the results to other healthcare settings, organizational cultures, or geographical contexts. Moreover, we recognize the need to deepen the differences between professional roles that, in terms of responsibilities and levels of autonomy, may shape the healthcare professional’s perception of leadership and organizational change. Future longitudinal studies involving multiple hospitals, more heterogeneous healthcare settings, and more balanced samples across occupational working group would help strengthen the generalizability and robustness of the findings.

The second is the performance and leadership variables measured with self‐report tools may increase the potential risk of common method bias. Therefore, future studies could adopt hetero evaluations and longitudinal research designs measured at different time points to exclude social desirability bias and causal relationships between variables. Furthermore, the use of a single‐item measure of GI could represent a limitation in effectively capture the construct. This indicator was selected to reduce respondent burden in the hospital setting since it has been widely validated and used in prior research [79, 87] and has demonstrated adequate reliability and validity across a range of social groups and contexts.

Third, any changes that occurred that could affect adaptive behaviors and change predisposition were not detected. In the future, it will be interesting to identify some particular changes that could affect the variables presented to better understand the effect of leadership style on adaptivity in a specific context of organizational changes.

Finally, it would be interesting to extend the study to job sectors other than healthcare to compare the results and because the model needs to be further explored.

## Author Contributions

The three authors contributed equally to the conceptualization and design, to the investigation, to the analysis and results interpretation, and to writing the original draft of the study. Alice Bonini also led the editing of the final version, wrote the review of the manuscript, and served as corresponding author.

## Funding

The authors have no financial or proprietary interests in any material discussed in this article. No funds, grants, or other support was received. Open access publishing facilitated by Universita degli Studi di Parma, as part of the Wiley ‐ CRUI‐CARE agreement.

## Disclosure

All authors read and approved the final version of the manuscript.

## Ethics Statement

Concerning the ethical approval, the research was carried out in accordance with the American Psychological Association (APA) and National Association of Psychology ethical standards for the treatment of human subjects and in accordance with the principles of the Declaration of Helsinki. Following the Italian requirements, ethics committee review is not legally mandated for survey‐based research when the following conditions are met: (1) participation is entirely voluntary, (2) participants are explicitly informed of their right to refuse or withdraw at any time without consequences, (3) no personal or sensitive data (as defined by GDPR Art. 9) are collected, and (4) data are fully anonymous at the point of collection, precluding any possibility of re‐identification. These conditions are grounded in Italian data protection law (D.lgs. 196/2003, as amended by D.lgs. 101/2018) and the EU General Data Protection Regulation (GDPR 2016/679), which distinguish between anonymous data—to which data protection obligations do not apply—and pseudonymous or identifiable data. In addition, the study was formally authorized by the hospital administration. Participants were healthcare professionals rather than patients, and their involvement was entirely unrelated to clinical care or treatment. Furthermore, the survey was administered online, and no identifying information was collected at any stage: responses were anonymous from the point of entry and could not be traced back to individual respondents. Finally, participation was fully voluntary; all participants were informed of the study’s purpose, its academic nature, the anonymity of their responses, and their right to decline or discontinue participation at any time without professional consequences. Data were accessible only to the research team and stored securely in accordance with GDPR requirements.

## Conflicts of Interest

The authors declare no conflicts of interest.

## Data Availability

The datasets generated during and analyzed during the current study are available from the corresponding author on reasonable request.

## References

[1] Alonso J. M. , Clifton J. , and Díaz-Fuentes D. , The Impact of New Public Management on Efficiency: Atn Analysis of Madrid’s Hospitals, Health Policy. (2015) 119, no. 3, 333–340, 10.1016/j.healthpol.2014.12.001. 25533550

[2] Rafferty A.E. and Griffin M.A. , Perceptions of Organizational Change: A Stress and Coping Perspective, Journal of Applied Psychology. (2006) 91, 1154–1162, 10.1037/0021-9010.91.5.1154. 16953776

[3] Limm H. , Anger]r P. , Heinmueller M. , Marten-Mittag B. , Nater U. M. , and Guendel H. , Self-Perceived Stress Reactivity Is an Indicator of Psychosocial Impairment at the Workplace, BMC Public Health. (2010) 10, no. 1, 10.1186/1471-2458-10-252. PMC2881886 20470413

[4] Nilsen P. , Seing I. , Ericsson C. , Birken S. A. , and Schildmeijer K. , Characteristics of Successful Changes in Health Care Organizations: An Interview Study With Physicians, Registered Nurses and Assistant Nurses, BMC Health Services Research. (2020) 20, no. 1, 10.1186/s12913-020-4999-8. PMC7045403 32106847

[5] Charbonnier-Voirin A. , El Akremi A. , and Vandenberghe C. , A Multilevel Model of Transformational Leadership and Adaptive Performance and the Moderating Role of Climate for Innovation, Group & Organization Management. (2010) 35, no. 6, 699–726, 10.1177/1059601110390833.

[6] Griffin M. A. , Neal A. , and Parker S. K. , A New Model of Work Role Performance: Positive Behavior in Uncertain and Interdependent Contexts, Australasian Marketing Journal. (2007) 50, no. 2, 327–347, 10.5465/amj.2007.24634438.

[7] Krijgsheld M. , Tummers L. G. , and Scheepers F. E. , Job Performance in Healthcare: A Systematic Review, BMC Health Services Research. (2022) 22, no. 1, 10.1186/s12913-021-07357-5. PMC8815187 35120495

[8] Park S. and Park S. , Employee Adaptive Performance and its Antecedents: Review and Synthesis, Human Resource Development Review. (2019) 18, no. 3, 294–324, 10.1177/1534484319836315.

[9] Allworth E. and Hesketh B. , Construct-Oriented Biodata: Capturing Change-Related and Contextually Relevant Future Performance, International Journal of Selection and Assessment. (1999) 7, no. 2, 97–111, 10.1111/1468-2389.00110.

[10] Pulakos E. D. , Schmitt N. , Dorsey D. W. , Arad S. , Borman W. C. , and Hedge J. W. , Predicting Adaptive Performance: Further Tests of a Model of Adaptability, Human Performance. (2002) 15, no. 4, 299–323, 10.1207/S15327043HUP1504_01.

[11] Pulakos E. D. , Arad S. , Donovan M. A. , and Plamondon K. E. , Adaptability in the Workplace: Development of a Taxonomy of Adaptive Performance, Journal of Applied Psychology. (2000) 85, no. 4, 612–624, 10.1037/0021-9010.85.4.612. 10948805

[12] Charbonnier-Voirin A. and Roussel P. , Adaptive Performance: A New Scale to Measure Individual Performance in Organizations: Adaptive Performace—A New Scale To Measure Individual Performance, Canadian Journal of Administrative Sciences. (2012) 29, no. 3, 280–293, 10.1002/cjas.232.

[13] Gordon H. J. , Demerouti E. , Le Blanc P. M. , Bakker A. B. , Bipp T. , and Verhagen M. A. M. T. , Individual Job Redesign: Job Crafting Interventions in Healthcare, Journal of Vocational Behavior. (2018) 104, 98–114, 10.1016/j.jvb.2017.07.002.

[14] Newton-Lewis T. , Munar W. , and Chanturidze T. , Performance Management in Complex Adaptive Systems: A Conceptual Framework for Health Systems, BMJ Global Health. (2021) 6, no. 7, 10.1136/bmjgh-2021-005582. PMC8323386 34326069

[15] Knutsen Glette M. , Ludlow K. , Wiig S. , Bates D. W. , and Austin E. E. , Resilience Perspective on Healthcare Professionals’ Adaptations to Changes and Challenges Resulting From the COVID-19 Pandemic: A Meta-Synthesis, BMJ Open. (2023) 13, no. 9, 10.1136/bmjopen-2023-071828. PMC10514639 37730402

[16] Faryal I. N. and Irum Naqvi , The Relationship Between Job Satisfaction and Job Performance: A Study on Frontline Healthcare Workers, ASSAP. (2023) 4, no. 1, 171–183, 10.52700/assap.v4i1.255.

[17] Braithwaite J. , Wears R. L. , and Hollnagel E. , Resilient Health Care: Turning Patient Safety on Its Head, International Journal for Quality in Health Care. (2015) 27, no. 5, 418–420, 10.1093/intqhc/mzv063. 26294709

[18] Anderson J. E. , Lavelle M. , and Reedy G. , Understanding Adaptive Teamwork in Health Care: Progress and Future Directions, Journal of Health Services Research & Policy. (2021) 26, no. 3, 208–214, 10.1177/1355819620978436. 33327787 PMC8182291

[19] Sanford N. , Lounsbury O. , Reedy G. , Rafferty D. A. M. , and Anderson J. E. , Team Adaptive Capacity and Adaptation in Dynamic Environments: A Scoping Review of the Literature, Human Factors in Healthcare. (2024) 6, 10.1016/j.hfh.2024.100089.

[20] Griffin M. A. , Parker S. K. , and Mason C. M. , Leader Vision and the Development of Adaptive and Proactive Performance: A Longitudinal Study, Journal of Applied Psychology. (2010) 95, no. 1, 174–182, 10.1037/a0017263. 20085414

[21] Bonini A. , Panari C. , Caricati L. , and Mariani M. G. , The Relationship Between Leadership and Adaptive Performance: A Systematic Review and Meta-Analysis, PLoS One. (2024) 19, no. 10, 10.1371/journal.pone.0304720. PMC11488715 39423211

[22] Bornman J. and Louw B. , Leadership Development Strategies in Interprofessional Healthcare Collaboration: A Rapid Review, JHL. (2023) 15, 175–192, 10.2147/JHL.S405983. 37641632 PMC10460600

[23] VanVactor J. D. , Collaborative Leadership Model in the Management of Health Care, Journal of Business Research. (2012) 65, no. 4, 555–561, 10.1016/j.jbusres.2011.02.021.

[24] Quinn R. E. , Applying the Competing Values Approach to Leadership: Toward an Integrative Framework, Leaders and Managers, 1984, Elsevier, 10–27, 10.1016/B978-0-08-030943-9.50010-3.

[25] Cameron K. S. and Quinn R. E. , Diagnosing and Changing Organisational Culture Based on Competing Values Framework, 2006.

[26] Lawrence K. A. , Lenk P. , and Quinn R. E. , Behavioral Complexity in Leadership: the Psychometric Properties of a New Instrument to Measure Behavioral Repertoire, Leadership Quarterly. (2009) 20, no. 2, 87–102, 10.1016/j.leaqua.2009.01.014.

[27] Fatima T. , Majeed M. , and Saeed I. , Does Participative Leadership Promote Innovative Work Behavior: The Moderated Mediation Model, BERC. (2017) 9, no. 4, 141–158, 10.22547/BER/9.4.7.

[28] McKimm J. , Ramani S. , Forrest K. et al., Adaptive Leadership During Challenging Times: Effective Strategies for Health Professions Educators: AMEE Guide No. 148, Medical Teacher. (2023) 45, no. 2, 128–138, 10.1080/0142159X.2022.2057288. 35543323

[29] Schaufeli W. B. , Salanova M. , González-romá V. , and Bakker A. B. , The Measurement of Engagement and Burnout: A Two Sample Confirmatory Factor Analytic Approach, Journal of Happiness Studies. (2002) 3, no. 1, 71–92, 10.1023/A:1015630930326.

[30] Maslach C. , Schaufeli W. B. , and Leiter M. P. , Job Burnout, Annual Review of Psychology. (2001) 52, no. 1, 397–422, 10.1146/annurev.psych.52.1.397. 11148311

[31] Schaufeli W. B. , Bakker A. B. , and Salanova M. , The Measurement of Work Engagement With a Short Questionnaire: A Cross-National Study, Educational and Psychological Measurement. (2006) 66, no. 4, 701–716, 10.1177/0013164405282471.

[32] Park Y. , Lim D. H. , Kim W. , and Kang H. , Organizational Support and Adaptive Performance: The Revolving Structural Relationships Between Job Crafting, Work Engagement, and Adaptive Performance, Sustainability. (2020) 12, 10.3390/su12124872.

[33] Breevaart K. , Bakker A. B. , Demerouti E. , Sleebos D. M. , and Maduro V. , Uncovering the Underlying Relationship Between Transformational Leaders and Followers’ Task Performance, Journal of Personnel Psychology. (2014) 13, no. 4, 194–203, 10.1027/1866-5888/a000118.

[34] Bakker A. B. , Demerouti E. , and Sanz-Vergel A. I. , Burnout and Work Engagement: the JD–R Approach, Annual Review of Organizational Psychology and Organizational Behavior. (2014) 1, 389–411, 10.1146/annurev-orgpsych-031413-091235.

[35] Bakker A. B. , Hakanen J. J. , Demerouti E. , and Xanthopoulou D. , Job Resources Boost Work Engagement, Particularly When Job Demands Are High, Journal of Educational Psychology. (2007) 99, no. 2, 274–284, 10.1037/0022-0663.99.2.274.

[36] Carasco-Saul M. , Kim W. , and Kim T. , Leadership and Employee Engagement: Proposing Research Agendas Through a Review of Literature, Human Resource Development Review. (2015) 14, no. 1, 38–63, 10.1177/1534484314560406.

[37] Decuypere A. and Schaufeli W. , Leadership and Work Engagement: Exploring Explanatory Mechanisms, German Journal of Human Resource Management. (2020) 34, no. 1, 69–95, 10.1177/2397002219892197.

[38] Al-Swidi A. K. , Mohd Nawawi M. K. , and Al-Hosam A. , Is the Relationship Between Employees’ Psychological Empowerment and Employees’ Job Satisfaction Contingent on the Transformational Leadership? A Study on the Yemeni Islamic Banks, ASS. (2012) 8, no. 10, 10.5539/ass.v8n10p130.

[39] Schaufeli W. B. , Engaging Leadership in the Job Demands-Resources Model, Career Development International. (2015) 20, no. 5, 446–463, 10.1108/CDI-02-2015-0025.

[40] Bass B. M. and Riggio R. E. , Transformational Leadership, 2006, Psychology Press, 10.4324/9781410617095.

[41] Okpala P. , Increasing Access to Quality Healthcare Through Collaborative Leadership, International Journal of Healthcare Management. (2020) 13, no. 3, 229–235, 10.1080/20479700.2017.1401276.

[42] Jundt D. K. , Shoss M. K. , and Huang J. L. , Individual Adaptive Performance in Organizations: A Review Adaptive Performance, Beyond Behavior. (2015) 36, no. S1, S53–S71, 10.1002/job.1955.

[43] Park S. and Park S. , How Can Employees Adapt to Change? Clarifying the Adaptive Performance Concepts, Human Resource Development Quarterly. (2021) 32, no. 1, 10.1002/hrdq.21411.

[44] Aguinis H. and Kraiger K. , Benefits of Training and Development for Individuals and Teams, Organizations, and Society, Annual Review of Psychology. (2009) 60, no. 1, 451–474, 10.1146/annurev.psych.60.110707.163505. 18976113

[45] Jetten J. , Haslam S. A. , Cruwys T. , Greenaway K. H. , Haslam C. , and Steffens N. K. , Advancing the Social Identity Approach to Health and Well‐Being: Progressing the Social Cure Research Agenda, European Journal of Social Psychology. (2017) 47, no. 7, 789–802, 10.1002/ejsp.2333.

[46] Turner J. C. , Brown R. J. , and Tajfel H. , Social Comparison and Group Interest in Ingroup Favouritism, European Journal of Social Psychology. (1979) 9, no. 2, 187–204, 10.1002/ejsp.2420090207.

[47] Caricati L. , Panari C. , and Melleri M. , Group Identification and Self‐Efficacy Associated With Quality of Life in Emergency Medical Services Volunteers: A Cross‐Sectional Investigation, Journal of Applied Social Psychology. (2020) 50, no. 8, 476–488, 10.1111/jasp.12675.

[48] Van Dick R. , Identification in Organizational Contexts: Linking Theory and Research From Social and Organizational Psychology, International Journal of Management Reviews. (2001) 3, no. 4, 265–283, 10.1111/1468-2370.00068.

[49] Haslam S. A. and Van Dick R. , A Social Identity Approach to Workplace Stress, Social Psychology and Organizations, 2011, Routledge, 357–384.

[50] Steffens N. K. , Haslam S. A. , Reicher S. D. et al., Leadership as Social Identity Management: Introducing the Identity Leadership Inventory (ILI) to Assess and Validate a Four-Dimensional Model, The Leadership Quarterly. (2014) 25, no. 5, 1001–1024, 10.1016/j.leaqua.2014.05.002.

[51] Tse H. H. M. and Chiu W. C. K. , Transformational Leadership and Job Performance: A Social Identity Perspective, Journal of Business Research. (2014) 67, no. 1, 2827–2835, 10.1016/j.jbusres.2012.07.018.

[52] Haslam S. A. , Reicher S. D. , and Platow M. J. , The New Psychology of Leadership: Identity, Influence and Power, 2020, 2nd edition, Routledge, 10.4324/9781351108232.

[53] Fransen K. , Haslam S. A. , Steffens N. K. , Vanbeselaere N. , De Cuyper B. , and Boen F. , Believing in Us: Exploring Leaders’ Capacity to Enhance Team Confidence and Performance by Building a Sense of Shared Social Identity, Journal of Experimental Psychology: Applied. (2015) 21, no. 1, 89–100, 10.1037/xap0000033. 25401268

[54] Men L. R. and Yue C. A. , Creating a Positive Emotional Culture: Effect of Internal Communication and Impact on Employee Supportive Behaviors, Public Relations Review. (2019) 45, no. 3, 10.1016/j.pubrev.2019.03.001.

[55] Schaufeli W. B. and Bakker A. B. , Job Demands, Job Resources, and Their Relationship With Burnout and Engagement: A Multi‐Sample Study, Journal of Organizational Behavior. (2004) 25, no. 3, 293–315, 10.1002/job.248.

[56] Bakker A. B. and Demerouti E. , The Job Demands‐Resources Model: State of the Art, Journal of Managerial Psychology. (2007) 22, no. 3, 309–328, 10.1108/02683940710733115.

[57] LePine J. A. , Colquitt J. A. , and Erez A. , Adaptability to Changing Task Contexts: Effects of General Cognitive Ability, Conscientiousness, and Openness to Experience, Personnel Psychology. (2000) 53, no. 3, 563–593, 10.1111/j.1744-6570.2000.tb00214.x.

[58] Ogunfowora B. , Bourdage J. , and Lee K. , Rater Personality and Performance Dimension Weighting in Making Overall Performance Judgments, Journal of Business and Psychology. (2010) 25, no. 3, 465–476, 10.1007/s10869-009-9144-y.

[59] Parker S. K. , Bindl U. K. , and Strauss K. , Making Things Happen: A Model of Proactive Motivation, Journal of Management. (2010) 36, no. 4, 827–856, 10.1177/0149206310363732.

[60] Akhtar R. , Boustani L. , Tsivrikos D. , and Chamorro-Premuzic T. , The Engageable Personality: Personality and Trait EI as Predictors of Work Engagement, Personality and Individual Differences. (2015) 73, 44–49, 10.1016/j.paid.2014.08.040.

[61] Inceoglu I. and Warr P. , Personality and Job Engagement, Journal of Personnel Psychology. (2011) 10, no. 4, 177–181, 10.1027/1866-5888/a000045.

[62] Petrou P. , Demerouti E. , and Schaufeli W. B. , Job Crafting in Changing Organizations: Antecedents and Implications for Exhaustion and Performance, Journal of Occupational Health Psychology. (2015) 20, no. 4, 470–480, 10.1037/a0039003. 25798717

[63] McMillan K. and Perron A. , Nurses Amidst Change: The Concept of Change Fatigue Offers an Alternative Perspective on Organizational Change, Policy, Politics, & Nursing Practice. (2013) 14, no. 1, 26–32, 10.1177/1527154413481811. 23549748

[64] Wang T. , Olivier D. F. , and Chen P. , Creating Individual and Organizational Readiness for Change: Conceptualization of System Readiness for Change in School Education, International Journal of Leadership in Education. (2023) 26, no. 6, 1037–1061, 10.1080/13603124.2020.1818131.

[65] Naami A. , Behzadi E. , Parisa H. , and Charkhabi M. , A Study on the Personality Aspects of Adaptive Performance Among Governmental Hospitals Nurses: A Conceptual Model, Procedia-Social and Behavioral Sciences. (2014) 159, 359–364, 10.1016/j.sbspro.2014.12.388.

[66] Di Fabio A. and Gori A. , Developing a New Instrument for Assessing Acceptance of Change, Frontiers in Psychology. (2016) 7, 10.3389/fpsyg.2016.00802. PMC4884749 27303356

[67] Quinn R. E. and Rohrbaugh J. , A Competing Values Approach to Organizational Effectiveness, Public Productivity Review. (1981) 5, no. 2, 10.2307/3380029.

[68] Balducci C. , Fraccaroli F. , and Schaufeli W. B. , Psychometric Properties of the Italian Version of the Utrecht Work Engagement Scale (UWES-9): A Cross-Cultural Analysis, European Journal of Psychological Assessment. (2010) 26, no. 2, 143–149, 10.1027/1015-5759/a000020.

[69] Leach C. W. , Van Zomeren M. , Zebel S. et al., Group-Level Self-Definition and Self-Investment: A Hierarchical (Multicomponent) Model of in-Group Identification, Journal of Personality and Social Psychology. (2008) 95, no. 1, 144–165, 10.1037/0022-3514.95.1.144. 18605857

[70] Kaya B. and Karatepe O. M. , Does Servant Leadership Better Explain Work Engagement, Career Satisfaction and Adaptive Performance Than Authentic Leadership?, IJCHM. (2020) 32, no. 6, 2075–2095, 10.1108/IJCHM-05-2019-0438.

[71] Eldor L. and Harpaz I. , A Process Model of Employee Engagement: The Learning Climate and Its Relationship With Extra‐Role Performance Behaviors, Journal of Organizational Behavior. (2016) 37, no. 2, 213–235, 10.1002/job.2037.

[72] Ali A. , Ali S. M. , and Xue X. , Motivational Approach to Team Service Performance: Role of Participative Leadership and Team-Inclusive Climate, Journal of Hospitality and Tourism Management. (2022) 52, 75–85, 10.1016/j.jhtm.2022.06.005.

[73] Kaltiainen J. and Hakanen J. , Fostering Task and Adaptive Performance Through Employee Well-Being: The Role of Servant Leadership, BRQ Business Research Quarterly. (2022) 25, no. 1, 28–43, 10.1177/2340944420981599.

[74] Busse R. and Regenberg S. , Revisiting the “Authoritarian Versus Participative” Leadership Style Legacy: A New Model of the Impact of Leadership Inclusiveness on Employee Engagement, Journal of Leadership & Organizational Studies. (2019) 26, no. 4, 510–525, 10.1177/1548051818810135.

[75] Chan S. C. H. , Participative Leadership and Job Satisfaction: The Mediating Role of Work Engagement and the Moderating Role of Fun Experienced at Work, LODJ. (2019) 40, no. 3, 319–333, 10.1108/LODJ-06-2018-0215.

[76] Buil I. , Martínez E. , and Matute J. , Transformational Leadership and Employee Performance: The Role of Identification, Engagement and Proactive Personality, International Journal of Hospitality Management. (2019) 77, 64–75, 10.1016/j.ijhm.2018.06.014.

[77] Van Dick R. , Lemoine J. E. , Steffens N. K. et al., Identity Leadership Going Global: Validation of the Identity Leadership Inventory Across 20 Countries, Journal of Occupational and Organizational Psychology. (2018) 91, no. 4, 697–728, 10.1111/joop.12223.

[78] Roberson Q. M. , Disentangling the Meanings of Diversity and Inclusion in Organizations, Group & Organization Management. (2006) 31, no. 2, 212–236, 10.1177/1059601104273064.

[79] Postmes T. , Haslam S. A. , and Swaab R. I. , Social Influence in Small Groups: an Interactive Model of Social Identity Formation, European Review of Social Psychology. (2005) 16, 1–42, 10.1080/10463280440000062.

[80] Kim E. and Glomb T. M. , Victimization of High Performers: The Roles of Envy and Work Group Identification, Journal of Applied Psychology. (2014) 99, no. 4, 619–634, 10.1037/a0035789. 24490964

[81] Yu C. and Frenkel S. J. , Explaining Task Performance and Creativity From Perceived Organizational Support Theory: Which Mechanisms Are More Important?, Journal of Organizational Behavior. (2013) 34, no. 8, 1165–1181, 10.1002/job.1844.

[82] Bakker A. B. and Demerouti E. , Cooper C. L. , Job Demands-Resources Theory, Wellbeing, 2014, John Wiley & Sons, Ltd, Chichester, UK, 1–28, 10.1002/9781118539415.wbwell019.

[83] Albrecht S. L. , Bakker A. B. , Gruman J. A. , Macey W. H. , and Saks A. M. , Employee Engagement, Human Resource Management Practices and Competitive Advantage: an Integrated Approach, Journal of Organizational Effectiveness, People and Performance. (2015) 2, no. 1, 7–35, 10.1108/JOEPP-08-2014-0042.

[84] Christian M. S. , Garza A. S. , and Slaughter J. E. , Work Engagement: A Quantitative Review and Test of Its Relations With Task and Contextual Performance, Personnel Psychology. (2011) 64, no. 1, 89–136, 10.1111/j.1744-6570.2010.01203.x.

[85] Chiaburu D. S. , Peng A. C. , Oh I.-S. , Banks G. C. , and Lomeli L. C. , Antecedents and Consequences of Employee Organizational Cynicism: A Meta-Analysis, Journal of Vocational Behavior. (2013) 83, no. 2, 181–197, 10.1016/j.jvb.2013.03.007.

[86] Liu H. , Jameel Ahmed S. , Anjum M. A. , and Mina A. , Leader Humility and Employees’ Creative Performance: The Role of Intrinsic Motivation and Work Engagement, Frontiers in Psychology. (2024) 15, 10.3389/fpsyg.2024.1278755. PMC10835991 38312395

[87] Doosje B. , Ellemers N. , and Spears R. , Perceived Intragroup Variability as a Function of Group Status and Identifcation, Journal of Experimental Social Psychology. (1995) 31, 410–436, 10.1006/jesp.1995.1018.

